# Inverted Pyramid Nanostructures Coupled with a Sandwich Immunoassay for SERS Biomarker Detection

**DOI:** 10.3390/nano15010064

**Published:** 2025-01-02

**Authors:** Wen-Huei Chang, Shao-Quan Zhang, Zi-Yi Yang, Chun-Hung Lin

**Affiliations:** 1 Department of Applied Chemistry, National Pingtung University, Pingtung 90003, Taiwan; 2 Department of Photonics, National Cheng Kung University, Tainan 70101, Taiwan; 3 Meta-nanoPhotonics Center, National Cheng Kung University, Tainan 70101, Taiwan; 4 Program on Key Materials, Academy of Innovative Semiconductor and Sustainable Manufacturing, National Cheng Kung University, Tainan 70101, Taiwan

**Keywords:** surface-enhanced Raman scattering (SERS), hyaluronic acid, biomarker detection, immunoassay, inverted pyramid, 4-mercaptobenzoic acid, malachite green

## Abstract

Cancer diagnostics often faces challenges, such as invasiveness, high costs, and limited sensitivity for early detection, emphasizing the need for improved approaches. We present a surface-enhanced Raman scattering (SERS)-based platform leveraging inverted pyramid SU-8 nanostructured substrates fabricated via nanoimprint lithography. These substrates, characterized by sharp apices and edges, are further functionalized with (3-aminopropyl)triethoxysilane (APTES), enabling the uniform self-assembly of AuNPs to create a highly favorable configuration for enhanced SERS analysis. Performance testing of the substrates using malachite green (MG) as a model analyte demonstrated excellent detection capabilities, achieving a limit of detection as low as 10^−12^ M. Building on these results, the SERS platform was adapted for the sensitive and specific detection of hyaluronic acid (HA), a key biomarker associated with inflammation and cancer progression. The system employs a sandwich immunoassay configuration, with substrates functionalized with antibodies to capture HA molecules and 4-MBA-labeled SERS tags for detection. This setup achieved an ultra-sensitive detection limit of 10^−11^ g/mL for HA. Comprehensive characterization confirmed the uniformity and reproducibility of the SERS substrates, while validation in complex biological matrices demonstrated their robustness and reliability, highlighting their potential in cancer diagnostics and biomarker detection.

## 1. Introduction

Cancer is a disease arising from pathological and physiological changes during cell division. It has emerged as a significant global health concern, leading to a substantial number of deaths annually. In 2020 alone, there were over 19.3 million newly diagnosed and reported cancer cases, resulting in approximately 10 million deaths [1,2,3]. The causes of cancer are multifaceted, including lifestyle factors such as poor diet, smoking, and sleep deprivation and environmental factors like pesticide residues, industrial chemicals, and air pollution, all of which contribute to the increasing cancer incidence. These trends underscore the need to develop effective treatments for various cancers and early detection biosensors for timely monitoring [4,5,6,7].

Advancements in science and medicine have revealed that numerous biomolecules play indispensable roles in regulating human physiological mechanisms, with studies indicating that these molecules are closely linked to various diseases, including cancer. For example, CYFRA21-1 fragments are detected in the serum of cervical cancer patients [8], while, in lung cancer patients, the squamous cell carcinoma antigen (SCCA) has been shown to correlate with tumor progression [9]. Alpha-fetoprotein (AFP) is typically found in the serum and serves as an early biomarker for hepatocellular carcinoma [10], and carcinoembryonic antigen (CEA) is overexpressed in cancers such as gastrointestinal cancers, breast cancer, and pancreatic cancer [11,12]. These molecules, known as biomarkers, serve as indicators of abnormalities and disease within the body. Detecting biomarkers not only facilitates the early diagnosis of pathological changes but is also instrumental in ongoing disease monitoring and prevention.

Currently, cancer detection and diagnosis involve a variety of methods, each with distinct advantages and limitations. Imaging techniques, such as X-ray, computed tomography (CT), magnetic resonance imaging (MRI), ultrasound, and positron emission tomography (PET) scans, are non-invasive and effective in locating and sizing tumors [13]. However, they often lack the sensitivity required for early-stage cancer detection and may expose patients to radiation. Histological and cytological methods, including biopsies and liquid biopsies, provide high specificity and definitive diagnoses through the direct analysis of tissue or cell samples [14]. However, these techniques are invasive, time-consuming, and may cause patient discomfort. Molecular and genetic testing, such as tumor marker analysis and genomic sequencing, offers precision by detecting genetic mutations and specific biomarkers, enabling targeted therapies [15]. Despite their accuracy, these methods can be costly and require specialized facilities. Immunological assays, including enzyme-linked immunosorbent assays (ELISA) and immunohistochemistry, are widely used for their sensitivity in detecting cancer-related proteins [16]. However, they are susceptible to background interference and may lack the sensitivity required to detect low-abundance biomarkers. Endoscopy provides a direct view of potential tumors and allows for sample collection, but it is invasive and may not be suitable for routine screening. Non-invasive screenings, such as stool tests, are convenient and accessible for population-wide screening but often lack the specificity and sensitivity required to confirm diagnoses.

While traditional methods remain essential in cancer diagnosis, they often face limitations, such as invasiveness, high costs, time inefficiency, and suboptimal sensitivity, particularly in early detection. Emerging technologies, such as optical sensing [17], address these challenges by offering sensitive, accurate, rapid, and non-invasive detection methods, making them valuable tools for the advancement of cancer diagnostics. Optical sensing detects changes in optical signals induced by biological interactions, generating outputs proportional to the concentration of the target analyte. This approach achieves high specificity by integrating biological recognition elements, such as enzymes, antibodies, aptamers, cells, or tissues, which are tailored to selectively bind to target analytes. This design enables the detection of a wide range of analytes, from small molecules to complex biomolecules. Supporting both label-free and labeled detection methodologies, optical sensing offers flexibility for diverse applications. Label-free detection measures the interaction between the target analyte and the recognition element by leveraging intrinsic changes in optical properties, such as absorption, transmission, reflection, phase, amplitude, or polarization [18,19]. Conversely, labeled detection employs tagging molecules, such as fluorophores [20,21], chemiluminescent agents, or Raman tags [22], to amplify signals and enhance the sensitivity, making it particularly effective for the detection of low-abundance analytes. By overcoming the limitations of traditional methods and enabling precise, rapid, and versatile biomarker detection, optical sensing represents a transformative approach in modern cancer diagnostics.

Surface-enhanced Raman scattering (SERS) [22,23,24] offers numerous advantages for the detection of molecules and biomarkers, making it a powerful tool in biomedical research and diagnostics. Its ultra-sensitive detection capability allows for the identification of biomarkers at extremely low concentrations, possibly even at the single-molecule level, which is critical for early disease diagnosis. SERS provides high specificity due to the unique molecular fingerprints of Raman spectra, enabling the precise identification of target analytes without interference from similar compounds. This technique is particularly advantageous in SERS-based immunoassays [22,25], where the high sensitivity and specificity of SERS are combined with the selectivity of antigen–antibody interactions. This allows for the robust detection of target biomarkers in complex biological samples, even at trace levels. Additionally, the multiplexing capability of SERS-based immunoassays enables the simultaneous detection of multiple biomarkers by using Raman-active tags with distinct spectral signatures, significantly improving the assay throughput and efficiency [25,26]. Unlike fluorescence-based methods, SERS is less susceptible to photobleaching and autofluorescence, making it well suited for biofluids and other challenging sample matrices. However, fluorescent analyte molecules or contaminants may still undergo photobleaching under intense laser exposure, so optimizing the laser power and exposure time is essential. As a non-destructive technique, SERS preserves the integrity of biological samples for further analysis. These advantages position SERS-based immunoassays as a cutting-edge approach for sensitive, specific, and multiplexed biomarker detection, with significant applications in early disease diagnosis, therapeutic monitoring, and personalized medicine.

Previously, we developed a non-invasive method for the detection of bladder cancer markers using gold nanomushrooms and sandwich immunoassays [18]. This approach relied on localized surface plasmon resonance (LSPR) refractive index sensing and provided a label-free detection platform. Building on this foundation, we aimed to enhance the sensitivity and specificity through a SERS-based detection system. The current study integrated antibody-functionalized substrates with Raman reporter molecules, specifically 4-mercaptobenzoic acid (4-MBA), enabling the precise detection of low-abundance analytes. To achieve this, we utilized an inverted pyramid SU-8 nanostructured substrate fabricated via nanoimprint lithography, a process known for its significant advantages in uniformity, reproducibility, and scalability for large-scale production. The distinct geometry of the inverted pyramids, with sharp apices and edges, enhances signal amplification, while the uniform distribution of the gold nanoparticles (AuNPs) self-assembled on the (3-aminopropyl)triethoxysilane (APTES)-functionalized surface creates a configuration that is highly conducive to SERS analysis. Additionally, the anisotropic properties of nanostructures have been shown in other studies to facilitate polarized SERS analysis by providing directionally dependent field enhancements that effectively couple with molecular vibrations, making it suitable for the detection of non-isotropic molecules [27]. Our substrates demonstrated excellent uniformity and reproducibility, highlighting the robustness of the fabrication method. Performance testing of the SERS substrate using malachite green (MG) as the analyte confirmed the robustness of the substrate and consistent signal enhancement capabilities. Hyaluronic acid (HA) was selected as the target biomarker due to its critical role in cancer progression and inflammation, making it a valuable indicator for early-stage disease detection. The platform employs a sandwich immunoassay configuration to ensure high specificity by leveraging the selective binding of antibodies to distinct HA epitopes. This study conducted a series of tests, including sensitivity and specificity evaluations, reproducibility assessments, and detection in complex biological matrices, to validate the performance of the platform. The findings highlight the ability of the SERS-based system to address the limitations of traditional cancer detection methods, such as invasiveness and low sensitivity.

## 2. Materials and Methods

### 2.1. Chemicals and Materials

Sodium citrate, isopropyl alcohol, and acetone were purchased from J.T. Baker (Avantor, Inc., Radnor, PA, USA). Gold (III) chloride trihydrate (HAuCl_4_·3H_2_O) and 1H,1H,2H,2H-perfluorodecyltrichlorosilane (F_13_-TCS) were obtained from Alfa Aesar (Ward Hill, MA, USA). Poly(methyl methacrylate) (PMMA ACRYREX^®^ CM-211) was supplied by CHIMEI (Tainan, Taiwan). Perfluoropolyether (PFPE)-urethane dimethacrylate (Fluorolink MD700) was provided by Solvay Specialty Polymers (Bollate, Italy). Potassium hydroxide (KOH) was sourced from Honeywell Fluka (Charlotte, NC, USA). Chromium etchant (CR-7) was purchased from KMG Electronic Chemicals (Fort Worth, TX, USA). SU-8 3050 was obtained from Kayaku Advanced Materials (Westborough, MA, USA). APTES (98%), 4-MBA, and 4-morpholinoethanesulfonic acid hydrate (MES) were purchased from Thermo Fisher Scientific (Waltham, MA, USA). MG, polyoxyethylenesorbitan monolaurate (TWEEN^®^ 20), 11-mercaptoundecanoic acid (MUA), 1-ethyl-3-(3-dimethylaminopropyl)carbodiimide (EDC), N-hydroxysuccinimide (NHS), bovine serum albumin (BSA), and HA sodium salt from rooster combs (H5388) were sourced from Sigma-Aldrich (St. Louis, MO, USA). Fetal bovine serum (FBS) was purchased from Hyclone (Cytiva, Marlborough, MA, USA). Sheep anti-hyaluronic acid antibody (anti-HA, 5029−9990, polyclonal) was supplied by Bio-Rad Laboratories (Hercules, CA, USA).

### 2.2. Nanoimprint Process

First, a PFPE mold replicating a silicon (Si) mold was produced (refer to reference [28,29] for details on PFPE mold fabrication and the nanoimprint process). The PFPE mold was aligned with a polymer-coated substrate, stacked, and placed into custom-built nanoimprint equipment, covered with a polyethylene terephthalate (PET) sheet. A compressed air press was then applied to the mold at a constant elevated temperature. Once the assembly had cooled to 40 °C, the PFPE mold was carefully peeled away, yielding patterned polymer nanostructures on the substrate.

### 2.3. Fabrication of Si Master Molds with Inverted Pyramid Nanostructures

**Scheme 1:**
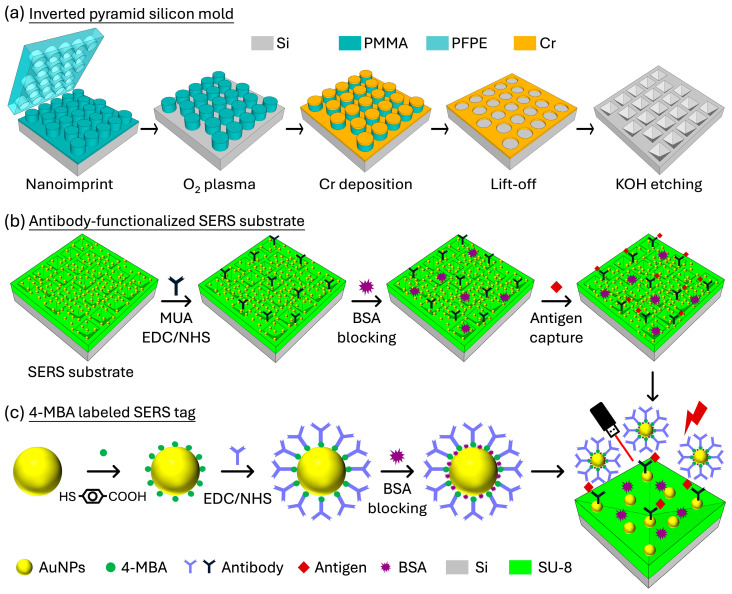
Schematic representation of key processes: (**a**) fabrication of inverted pyramid nanostructures on a Si substrate, (**b**) preparation of an antibody-functionalized SERS substrate for antigen capture, and (**c**) preparation of SERS tags by conjugating 4-MBA and antibodies to AuNPs for signal detection.

The fabrication process of Si master molds with inverted pyramid nanostructures is depicted in Scheme 1a. First, a 200-nm-thick PMMA layer was spin-coated onto a Si substrate and baked at 160 °C for 20 min. A PFPE mold featuring a hexagonal nanohole array was then prepared, followed by a nanoimprint process at 3 bar pressure for 10 min at a constant temperature of 120 °C. This resulted in the formation of a PMMA nanopillar array on the Si substrate. The residual PMMA layer was subsequently removed using oxygen plasma etching. A 60 nm chromium (Cr) film was then deposited onto the patterned resist via electron-beam evaporation, and a Cr hole array mask was produced through an acetone lift-off process.

To prepare the Si etchant, 65 mL of deionized water was heated to 60 °C in a glass crystallizing dish on a hot plate. Afterward, 32.2 g of KOH pellets was added and allowed to dissolve completely, with the solution reaching thermal equilibrium at 60 °C for 10 min. Next, 10 mL of isopropanol was introduced to the etching solution and stirred with a magnetic stirrer to slow the Si etching rate. The final KOH concentration was approximately 30.7 wt%. The Si substrate with the Cr hole array mask was immersed in this KOH solution, and the etching solution was agitated for 3 min with a magnetic stirrer to remove hydrogen bubbles on the Si surface. Following etching, the substrate was rinsed with deionized water and dried with nitrogen flow. The sample was then immersed in a glass crystallizing dish containing the Cr etchant and agitated in an ultrasonic cleaner for 10 min to remove the Cr mask, completing the fabrication of the inverted pyramid Si master mold.

### 2.4. Synthesis of AuNPs

AuNPs were synthesized using the Turkevich method [18,30]. In a 250 mL three-necked flask, 150 mL of a 2.2 mM sodium citrate solution in deionized water was added and stirred vigorously with a magnetic stirrer in a silicone oil bath. A condenser was connected to prevent solvent evaporation. Once the solution reached boiling, 1 mL of a 25 mM HAuCl_4_ solution was introduced, and the mixture was stirred for 15 min. During this time, the solution color transitioned from transparent to yellow, followed by dark bluish gray, and finally to pink, indicating the formation of AuNP seeds. The solution was subsequently cooled to 85 °C, as measured by a mercury thermometer. This process was repeated twice more by adding 1 mL of 25 mM HAuCl_4_ each time and stirring for 30 min to promote further nanoparticle formation.

To further grow the AuNPs to a larger size, 50 mL of the AuNP solution was removed from the flask and replaced with 50 mL of deionized water and 2 mL of a 60 mM sodium citrate solution. After 2 min, another 1 mL of 25 mM HAuCl_4_ was added, and the reaction continued for 30 min. A further 1 mL of 25 mM HAuCl_4_ was added, with the reaction proceeding for an additional 30 min. This process was repeated multiple times to achieve the desired AuNP size. Finally, the solution was cooled to room temperature and stored at 4 °C for future experimentation.

### 2.5. Self-Assembled AuNPs on APTES-Functionalized Nanostructured Surface

A Si substrate was prepared by spin coating with an SU-8 solution (20 μL of SU-8 3050 in cyclopentanone, 1:7 weight ratio) at 500 rpm for 5 s, followed by 3000 rpm for 25 s. Soft baking was then performed at 95 °C for 10 min, producing a layer that was approximately 327 nm thick. A PFPE mold was fabricated from a Si master mold featuring inverted pyramid nanostructures. The nanoimprinting process was conducted at 80 °C, applying pressure of 3 bar for 7 min. After cooling to 40 °C, the mold–substrate stack was exposed to UV irradiation (1 min at 39.6 mW/cm^2^, 14.7 cm distance) to cure the SU-8 layer. Following irradiation, the PFPE mold was carefully removed, yielding SU-8 inverted pyramid nanostructures.

To hydroxylate the SU-8 surface, the sample underwent UV–ozone treatment for 30 min. The sample was then immersed in 10 mL of deionized water containing 53 μL of APTES (0.5 wt%) and heated on a preheated hotplate at 75 °C for 1 h. After rinsing with deionized water and drying, the sample was transferred to a 12-well plate, where 800 μL of deionized water and 200 μL of AuNP solution were added. The sample was left undisturbed for 12 h, allowing the self-assembly of the AuNPs on the nanostructured SU-8 surface. Unbound AuNPs were subsequently rinsed off with deionized water. A post-bake at 95 °C was performed to enhance AuNP adhesion, completing the fabrication of the SERS substrate.

### 2.6. Antibody Functionalized to the SERS Substrate

The SERS substrate was immersed in a 20 mM MUA solution and agitated at 120 RPM for 12 h to promote Au-S bond formation. Afterward, the substrate was thoroughly rinsed with alcohol and dried. A mixture of 150 mM EDC and NHS in MES buffer (pH 6.3) was prepared at a 1:1.5 volume ratio, and 50 μL of this solution was applied to the SERS substrate. The substrate was shaken at 120 RPM for 30 min and then rinsed with deionized water and dried.

Following activation, 50 μL of a 10 μg/mL anti-HA solution was applied to the substrate, which was shaken at 120 RPM for 2 h. The substrate was subsequently rinsed with deionized water and dried. Finally, 50 μL of a 0.1% BSA solution was added to the substrate; it was shaken at 120 RPM for 30 min to block non-specific binding sites and then rinsed with deionized water and dried, completing the functionalization process.

### 2.7. Preparation of 4-MBA-Labeled SERS Tags

A solution was prepared by combining 1 μL of TWEEN 20, 10 μL of a 0.1 mM 4-MBA solution, and 990 μL of AuNPs in a centrifuge tube. The tube was rotated at 40 rpm for 12 h to allow the thorough modification of the AuNPs with the Raman reporter molecule 4-MBA. The 4-MBA-labeled AuNPs were then centrifuged at 6000 rpm for 10 min. After discarding the supernatant, the labeled AuNPs were resuspended in 1 mL of 10 mM MES buffer.

Then, a mixture of 350 mM EDC and NHS in MES buffer was prepared at a 1:1.5 volume ratio. To activate the 4-MBA-labeled AuNP surface, 10 μL of this EDC/NHS solution was added to the AuNP suspension and it was mixed on a shaker for 30 min. Subsequently, 10 μL of an anti-HA solution (10 μg/mL) was added, followed by an additional 2 h of mixing. To block non-specific binding sites, 10 μL of 0.1% BSA solution was added and the sample was mixed for another 30 min. Finally, the 4-MBA-labeled SERS tags were centrifuged at 5500 rpm for 10 min, the supernatant was discarded, and the SERS tags were resuspended in 1 mL of deionized water.

### 2.8. Sandwich Immunoassay for HA Molecule Detection

A 50 μL solution of HA at a specific concentration was applied to the anti-HA functionalized SERS substrate and it was incubated on a shaker at 120 rpm for 2 h. After incubation, the substrate was rinsed with deionized water and dried using a nitrogen stream. Subsequently, 50 μL of 4-MBA-labeled SERS tags were added to the antigen–antibody-functionalized SERS substrate and it was incubated on a shaker for 4 h. Following the reaction, the substrate was again rinsed with deionized water and dried with a nitrogen stream, after which it was ready for detection.

### 2.9. Characterization

Raman measurements were performed using a modular Raman system (HORIBA iHR320, Kyoto, Japan) equipped with a 632.8 nm He-Ne laser and an Olympus BX53 optical microscope (Tokyo, Japan). Raman signals were collected through a 20× objective lens with a 5 s integration time. The laser beam delivered to the sample was unpolarized, with measured power of 1.9 mW after transmission through the lens. Unless otherwise specified, all temperature values reported in this study were measured using a thermocouple (T-60, RiXEN, New Taipei, Taiwan). Scanning electron microscopy (SEM) images were captured using a field emission scanning electron microscope (SU8000, Hitachi, Tokyo, Japan). Transmission electron microscopy (TEM) images were obtained with a JEM-2100F CS STEM (JEOL, Tokyo, Japan). X-ray photoelectron spectroscopy (XPS) analysis was performed using a PHI 5000 VersaProbe (ULVAC-PHI, Chigasaki, Japan). The particle size distribution and zeta potential of the AgNPs were measured using a dynamic light scattering (DLS) analyzer (SZ-100, HORIBA, Kyoto, Japan). The absorption spectrum was measured by employing a microplate reader (Infinite M200 PRO, TECAN, Männedorf, Switzerland). A Mie scattering simulation was performed using the MiePlot v4.6.21 software [31].

## 3. Results and Discussion

### 3.1. Fabrication of SU-8 Inverted Pyramid Nanostructures

**Figure 1:**
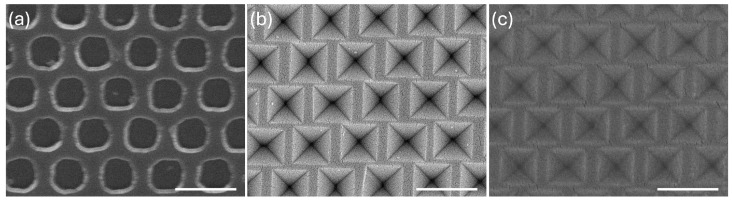
Fabrication of inverted pyramid nanostructures. (**a**) Cr hole array mask with 400 nm period and 265 nm hole size remains on the substrate after lift-off. (**b**) Inverted pyramid Si structures, approximately 285 nm in size, formed through KOH anisotropic wet etching. (**c**) SU-8 inverted pyramid nanostructures imprinted on a Si substrate using a PFPE mold replicated from the Si master in (**b**). Scale bars in (**a**–**c**) represent 500 nm.

The process of etching inverted pyramid structures on a Si substrate involves nanoimprint lithography followed by wet etching, utilizing the anisotropic etching properties of Si. A schematic of the fabrication process is shown in Scheme 1a. Initially, a PMMA layer was applied to the Si surface, and nanoimprinting was used to define the area for a patterned hard mask. A layer of Cr hard mask was then deposited to protect the regions that would not undergo etching. The subsequent lift-off process removed the PMMA, leaving a Cr hole array hard mask, as illustrated in Figure 1a. The period of the hole array was 400 nm, and the hole size was approximately 265 nm. Subsequently, the Si substrate was etched to create the inverted pyramid nanostructures using a KOH solution for wet etching. The Si etch rate varied along the crystallographic directions, with faster etching along the <100> direction than along the <111> direction. This anisotropic etching produced a distinctive inverted pyramid structure, with the depth and size controlled by the etching time and the dimensions of the patterned Cr openings. Finally, the wafer was cleaned, and any remaining Cr mask was removed, revealing well-defined inverted pyramids that were approximately 285 nm in size, as shown in Figure 1b. The Si substrate with these inverted pyramid nanostructures served as a master mold, which was then replicated into a PFPE working mold. This PFPE mold was used to imprint an SU-8 thin film on another Si substrate, forming SU-8 inverted pyramid nanostructures on the Si substrate, as shown in Figure 1c.

### 3.2. APTES-Directed Self-Assembly of AuNPs on SU-8 Inverted Pyramids for SERS Substrate

**Figure 2:**
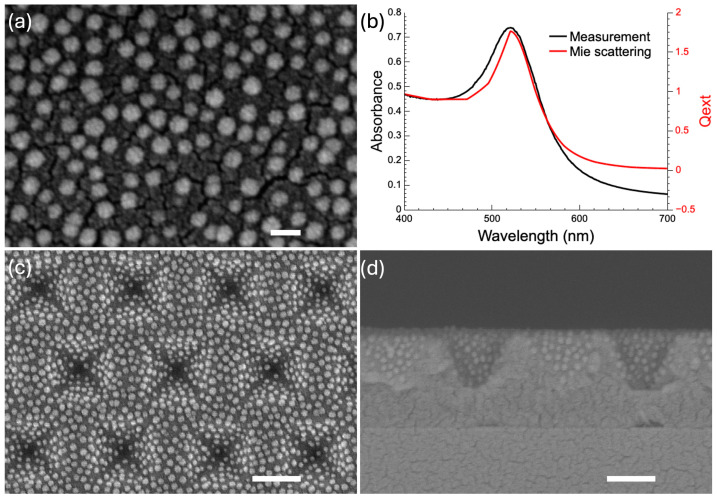
Functionalization and AuNP attachment on SU-8 inverted pyramid nanostructures. (**a**) SEM image of synthesized AuNPs with an average size of approximately 24.6 nm, self-assembled on an APTES-functionalized SU-8 film. (**b**) Absorption spectrum of the AuNP solution, displaying a peak near 521 nm, alongside Mie scattering simulation results that indicate a corresponding AuNP size of 25.1 nm for the same absorption peak wavelength. (**c**) Top-view and (**d**) cross-sectional SEM images of AuNPs self-assembled on the APTES-functionalized SU-8 inverted pyramids. Scale bars in (**a**,**c**,**d**) represent 50 nm and 200 nm, respectively.

We functionalized the surfaces of the SU-8 inverted pyramid nanostructures with APTES to facilitate AuNP attachment. The AuNPs were synthesized using the Turkevich method [18,30], with sodium citrate as the reducing agent. As shown in the SEM image in Figure 2a, the synthesized AuNPs had an average size of approximately 24.6 nm, estimated using the ImageJ v1.53 software. The AuNP solution exhibited a strong wine-red color, and its absorption spectrum, shown in Figure 2b, displayed a peak near 521 nm. For the absorption spectrum measurement, the AuNP solution (300 μL) was placed in a single well of a 96-well plate, with an approximate liquid depth of 9.67 mm. A comparison with the Mie scattering simulation results indicates that the simulated AuNPs, which had the same absorption peak wavelength, had a size of 25.1 nm—closely aligning with the size estimated from the SEM images. In the simulation, the AuNPs were modeled as uniformly sized spherical particles, evenly distributed in an aqueous solution. However, actual synthesized AuNPs may exhibit some size variation. Despite this, the discrepancy between the simulated and actual particle sizes remains within an acceptable range.

The SU-8 inverted pyramid substrate was treated in a UV–ozone environment to introduce hydroxyl groups (-OH) onto its surface. Subsequently, the substrate was immersed in a 0.5 wt% APTES solution, producing an amine-functionalized surface (-NH_2_). In a solution environment, the -NH_2_ groups on the substrate surface could become protonated to -NH_3_^+^, thereby imparting a positive charge to the surface. Since the AuNPs synthesized with sodium citrate carried a negative charge, immersing the positively charged substrate in the AuNP solution facilitated their electrostatic attachment to the SU-8 inverted pyramid surface through ionic interactions. Figure 2c,d present the top and cross-sectional SEM images of the AuNPs self-assembled on the APTES-functionalized SU-8 nanostructures. The AuNPs were uniformly distributed across the surfaces of the inverted pyramid nanostructures. The sharp apices and edges of the inverted pyramids, coupled with the even distribution of the AuNPs, formed a configuration that was highly conducive to enhanced SERS analysis. The cross-sectional SEM image captured the sample along a randomly fractured plane, which did not pass through the pyramid tips, giving the inverted pyramids a smaller appearance in this view. This substrate was designed for use in subsequent SERS applications.

### 3.3. Performance Testing of SERS Substrate Using MG as Analyte

**Figure 3:**
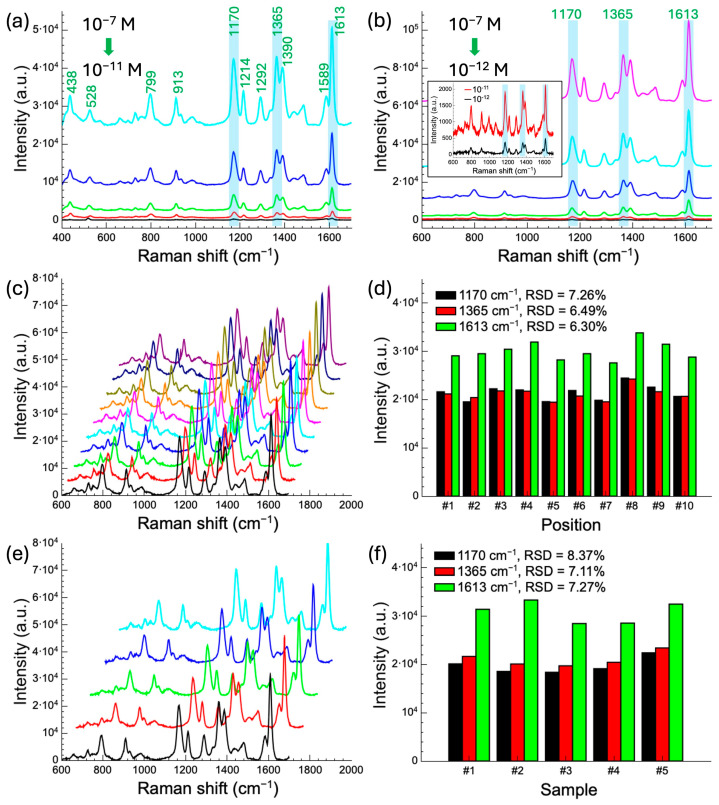
Evaluation of SERS substrate performance using MG as the test analyte. (**a**) SERS spectra of MG at concentrations of 10^−7^ to 10^−11^ M, with the average intensity from five locations per sample. (**b**) SERS spectra of MG on an F_13_-TCS-treated hydrophobic substrate, ranging from 10^−7^ to 10^−12^ M, with an inset showing spectra at 10^−11^ and 10^−12^ M. (**c**) SERS spectra of 10^−7^ M MG measured at 10 different locations along the coffee ring on a single substrate. (**d**) Signal intensities at 1170, 1365, and 1613 cm^−1^ for these points, with RSDs of 7.26%, 6.49%, and 6.30%. (**e**) SERS spectra at 10^−7^ M MG from five different substrates. (**f**) Signal intensities at the same peaks across substrates, with RSDs of 8.37%, 7.11%, and 7.27%.

MG, one of the most commonly used molecules for the testing of SERS substrates due to its strong and well-defined Raman spectral peaks, was used as the test analyte to evaluate the performance of our SERS system. A 10 μL volume of MG solution, prepared at varying concentrations, was dropped onto a SERS substrate and dried under vacuum conditions. Figure 3a displays the SERS spectra of MG across a concentration range from 10^−7^ to 10^−11^ M, with each spectrum representing the average intensity at each concentration, measured at five different locations per sample. Key peaks in the spectra include the characteristic peak at 1170 cm^−1^, corresponding to the in-plane ring bending vibration of the C-H bond; the peak at 1365 cm^−1^ due to the symmetric stretching of the N-phenyl bond; and the peak at 1613 cm^−1^ associated with the C-C stretching vibration within the benzene ring. Detailed peak assignments are summarized in Table 1 [32,33,34]. The background SERS spectrum from a blank nanostructured substrate, shown in Figure S1 (Supporting Information, Note S1), reflects the intrinsic signal from substrate–nanoparticle interactions without the analyte.

To further enhance the sensitivity of the system, we treated the surface of the substrate with F_13_-TCS to impart hydrophobic properties [24]. This treatment increased the contact angle between the analyte droplet and the surface of the SERS substrate, promoting a coffee-ring effect that concentrated the MG within a smaller area and increased its density per unit area. This modification improved the detection capabilities of the SERS substrate at lower concentrations. Figure 3b displays the SERS spectra of MG on the F_13_-TCS-treated substrate, covering a concentration range of 10^−7^ to 10^−12^ M. The hydrophobicity of the substrate enhanced the analyte concentration and sensitivity, enabling the detection limit of MG to reach 10^−12^ M, as highlighted in the inset figure. Notably, even at this ultra-low concentration, the three characteristic peaks remained distinct and clearly visible. To quantify the SERS enhancement provided by the substrate, the analytical enhancement factor (AEF) was calculated for the 1613 cm^−1^ peak as 8.10 × 10^7^ using the following formula:

(1) $$AEF=\frac{I_{SERS}/C_{SERS}}{I_{Raman}/C_{Raman}}$$

where *I_SERS_* and *C_SERS_* are the SERS intensity (469.31) and the concentration of MG (10^−12^ M) measured on the SERS substrate, while *I_Raman_* and *C_Raman_* are the Raman intensity (579.29) and the concentration of MG (10^−4^ M) measured on a bare Si substrate without AuNPs.

We further evaluated the uniformity and reproducibility of detection on the SERS substrate. Figure 3c shows the SERS spectra of MG at a concentration of 10^−7^ M, measured at 10 distinct locations along the coffee ring on the same substrate. Figure 3d presents the intensities at 1170, 1365, and 1613 cm^−1^ for these 10 points. The relative standard deviations (RSDs, calculated as the ratio of the standard deviation to the mean from the signal intensities of these 10 points) are 7.26%, 6.49%, and 6.30%, respectively, indicating good uniformity along the coffee-ring area. Figure 3e displays the SERS spectra measured on five different substrates, with the signal intensities at the same three characteristic peaks shown in Figure 3f. The RSD values for reproducibility across substrates were 8.37%, 7.11%, and 7.27%, respectively. These results demonstrate the excellent structural uniformity and even distribution of the AuNPs on the SERS substrate, and the consistent signal intensities across the different samples highlight the stability of the fabrication process.

### 3.4. Application of SERS Substrate in HA Molecule Detection

In addition to the environmental detection of MG molecules, the SERS substrate can also be applied in the biomedical field, particularly in SERS-based immunoassay techniques, which have gained popularity in recent years. In this study, we aimed to modify HA molecules with 4-MBA, a compound containing an aromatic ring structure. Although 4-MBA and MG are distinct compounds, they share the common feature of aromatic rings, which contribute to their characteristic SERS peaks. By employing 4-MBA as the Raman tag, we utilized its distinct SERS signals to achieve highly sensitive detection in our system.

Notably, HA molecules are closely associated with inflammation and cancer cell metastasis, with their concentrations in bodily fluids significantly increasing as cancer progresses [35]. Currently, HA levels are considered a clinical marker for malignancy, making HA a potential indicator for the early screening of cancer and inflammation, such as bladder cancer [36,37,38], breast cancer [39], upper gastrointestinal cancer [40], prostate cancer [41], and liver fibrosis in chronic liver disease [42].

In bladder cancer, the distribution of high-molecular-weight HA (HMW-HA) and low-molecular-weight HA (LMW-HA) varies with the tumor grade, each serving distinct physiological roles [36,43]. In low-grade (G1) bladder cancer, urinary HA primarily consists of HMW-HA (~10^6^ Daltons) and small angiogenic HA fragments. During the early stages, HMW-HA provides structural stability, forming a protective barrier around tumor cells that shields them from immune surveillance and supports their survival. This barrier minimizes immune attacks and creates a favorable environment for tumor growth. As bladder cancer progresses to the intermediate (G2) and high-grade (G3) stages, both HMW-HA and small HA fragments are found in the urine, reflecting changes in HA dynamics [36,44]. HMW-HA facilitates tumor cell adhesion and migration, driving invasion and metastasis, while LMW-HA fragments, generated by hyaluronidase activity, stimulate angiogenesis to promote tumor growth. The balance between HMW-HA and LMW-HA, regulated by hyaluronidase activity, is a key factor in bladder cancer progression [36,45].

HMW-HA is notable for its structural stability, aiding immune evasion, and increasing drug resistance [43,45]. Compared to LMW-HA, it more accurately reflects abnormalities in the tumor microenvironment. Urinary HA concentration measurements show HMW-HA’s diagnostic effectiveness, achieving sensitivity of 91.9% and specificity of 92.8%, making it a highly reliable non-invasive biomarker for diagnosis and disease monitoring [36]. Additionally, HMW-HA’s anti-inflammatory properties reduce inflammation in the tumor microenvironment, further enhancing its utility as a biomarker. In our study, the nominal weight-average MW of the HA obtained from Sigma Aldrich was approximately 1.5–1.6 × 10^6^ Da [46], closely aligning with the characteristics of HMW-HA reported in previous studies.

Furthermore, the HA used in medical esthetics shares the same molecular formula, (C_14_H_21_NO_11_)_n_. Consequently, patients who have previously received HA injections may present altered HA levels in their bodily fluids, potentially leading to misinterpretation during early cancer screenings. According to the literature [47], HA injections are contraindicated for certain individuals, including pregnant or breastfeeding women, children, individuals with abnormal liver or kidney function, those with major health conditions, individuals with clotting disorders or on anticoagulants due to an increased risk of bleeding, and individuals with cancer or a history of cancer, as some studies suggest that HA may promote cancer cell growth. Therefore, medical evaluation is typically conducted before HA injections to mitigate potential risks and avoid misinterpretation in subsequent diagnostic screenings.

To achieve the sensitive and specific detection of HA molecules, we developed a SERS-based sandwich immunoassay, a method commonly used in ELISA for biochemical analysis. Utilizing the previously described SERS substrate, we tailored the approach to detect HA molecules. In earlier experiments with MG molecules, an F_13_-TCS surface treatment was applied to the SERS substrate to enhance the hydrophobicity, thereby reducing the minimum detectable concentration. However, for HA detection, antibodies were conjugated directly onto the self-assembled AuNPs on the surface of the SERS substrate to enable specific binding to the target biomarker. To retain the functional groups required for antibody conjugation, the hydrophobic treatment was omitted.

#### 3.4.1. Preparation of Antibody-Functionalized SERS Substrates and 4-MBA-Labeled SERS Tags

Scheme 1b,c illustrate the preparation of the antibody-functionalized SERS substrates and the SERS tags, respectively, for biomarker detection. This detection process involves a sandwich immunoassay configuration that requires two antibodies: one for the capture of the target antigen and another for signal detection. To ensure high specificity, polyclonal antibodies were employed, allowing the capture and detection antibodies to bind to distinct epitopes on the HA molecule, the target biomarker. In this approach, the capture antibodies are immobilized on the SERS substrate, providing a stable and efficient platform for the binding of HA molecules. The detection antibodies are conjugated with the Raman reporter molecule 4-MBA, which produces a strong and unique Raman signal. In the presence of HA molecules, they are sandwiched between the capture antibodies anchored on the substrate and the detection antibodies attached to the Raman tags. This sandwich configuration ensures the precise recognition of HA molecules and significant amplification of the Raman signal, enabling highly sensitive and accurate detection. By combining the specificity of antibodies, the signal-enhancing properties of the Raman tag, and the robustness of the antibody-functionalized SERS substrate, this method offers a powerful and reliable approach for the effective detection of HA molecules.

To effectively capture HA molecules, we first functionalized the substrate with anti-HA antibodies. Anti-HA, a protein containing multiple amino groups (-NH_2_), was conjugated to the substrate using EDC/NHS coupling agents to activate the carboxyl groups (-COOH) on MUA. This activation facilitated the formation of stable amide bonds between the carboxyl groups on MUA and the amino groups on anti-HA. MUA, which features both carboxyl and thiol (-SH) functional groups, securely binds to AuNPs via strong Au-S bonds, ensuring the efficient and stable immobilization of anti-HA on the SERS substrate. The experimental parameters, such as the antibody concentration, immobilization time, and antibody–antigen interaction time, were optimized based on our previous research [18]. Changes in sulfur (S) and nitrogen (N) content during the antibody modification on the SERS substrate were analyzed using XPS, as shown in Figure S2 (Supporting Information, Note S2). Figure S2a shows that the blank SERS substrate does not contain sulfur and only has a faint nitrogen signal. After applying APTES to generate positively charged amino groups (-NH_2_) on the AuNPs for ionic bonding and immersion in MUA to form Au-S bonds, a sulfur signal appears, as shown in Figure S2b. Subsequently, activating the carboxyl groups of MUA with EDC/NHS and binding them with the amino groups (-NH_2_) on anti-HA produces a clearer nitrogen signal, as shown in Figure S2c. To prevent potential interference during HA capture caused by unreacted carboxyl groups not conjugated with anti-HA, BSA was used to block these residual sites. This blocking step minimized non-specific binding and ensured the integrity of the SERS substrate for HA capture. Subsequently, the HA capture by the antibody-conjugated SERS substrate was completed through the specific binding between the antibodies and antigens.

To prepare Raman tags for signal detection, we conjugated 4-MBA to AuNPs with an approximate diameter of 37.4 nm. It is known that 4-MBA contains a thiol (-SH), benzene ring and carboxyl (-COOH) groups. At high concentrations, its short chain length can lead to unstable bonding with AuNPs, resulting in aggregation. To mitigate this, we added a small amount of TWEEN 20 to the AuNP solution, which effectively increased the distance between negatively charged particles and reduced aggregation. The thiol group of 4-MBA forms a covalent bond with gold via an Au-S linkage, while the carboxyl group is activated using EDC/NHS chemistry to facilitate binding with anti-HA antibodies. Subsequently, BSA was used to block any remaining vacant sites on the gold surface, preventing non-specific binding. This preparation ensures the stability and specificity of the Raman tags for subsequent detection applications. Details regarding the optimal 4-MBA concentration, EDC/NHS concentration, AuNP size, and sandwich assembly time for the preparation of 4-MBA-labeled SERS tags are provided in the Supporting Information (Notes S3 and S4).

**Figure 4:**
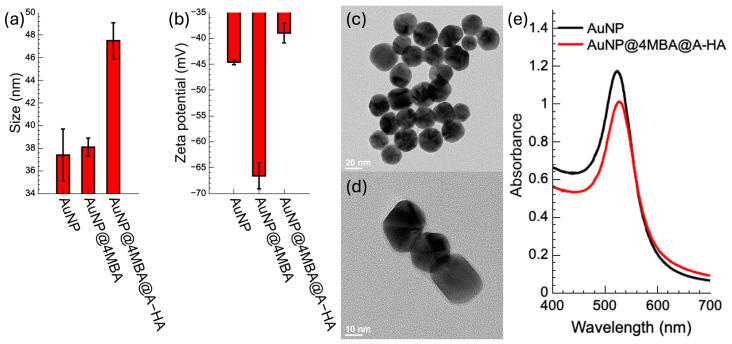
(**a**,**b**) DLS measurements of particle size and zeta potential variations after sequential modifications with 4-MBA and anti-HA antibodies. (**c**,**d**) TEM images showing morphological changes in AuNPs before and after conjugation with 4-MBA and anti-HA antibodies. (**e**) Absorption spectrum showing a redshift of 3.8 nm after anti-HA modification.

We analyzed changes in the AuNPs before and after modification with 4-MBA and anti-HA. Figure 4a shows the DLS measurements, demonstrating an increase in particle size from approximately 37.4 nm to 47.5 nm, indicating growth of about 10.1 nm. This observed increase aligns with our expectations, suggesting the successful conjugation of 4-MBA and anti-HA antibodies to the AuNPs. Such an increase in the hydrodynamic diameter is consistent with the formation of a molecular layer on the nanoparticle surface. Figure 4b highlights the zeta potential variations of the AuNPs at each stage of modification with 4-MBA and the anti-HA antibodies. The observed changes in the zeta potential can be attributed to the surface chemistry modifications of the AuNPs. After conjugation with 4-MBA, the zeta potential becomes more negative due to the introduction of negatively charged carboxyl groups (-COOH) from 4-MBA, which are exposed on the nanoparticle surface. This increases the surface charge density, resulting in a lower zeta potential. Following the conjugation of the anti-HA antibodies, the zeta potential rises. This is due to the protein nature of anti-HA, which contains positively charged amino groups (-NH_2_) that partially neutralize the negative charge on the surface. Additionally, the large size of the antibody creates a spatial shielding effect, reducing the overall negative charge density on the nanoparticle surface. Furthermore, the covalent binding between anti-HA and the carboxyl groups of 4-MBA (via EDC/NHS activation) consumes some of the negative charges, further contributing to the increase in zeta potential. Despite these modifications, the particles retained their negative charge and showed no signs of aggregation, demonstrating the stability and successful functionalization of the nanoparticles. These changes in zeta potential provide clear evidence of the stepwise surface modifications and the associated charge redistribution, confirming the effectiveness of the functionalization process. In Figure 4c,d, TEM images clearly reveal the morphological changes in the AuNPs after 4-MBA and antibody modification. A distinct halo surrounding the nanoparticles is observed, indicating successful surface functionalization with the anti-HA antibodies. Additionally, the absorption spectrum of the AuNPs, as shown in Figure 4e, exhibits a redshift of 3.8 nm following anti-HA modification. This shift, detected using a full-wavelength enzyme immunoassay analyzer, further confirms the successful conjugation of the anti-HA antibodies to the nanoparticle surface, reflecting changes in the local refractive index due to the formation of a molecular layer. Together, these results validate the effective surface modification of the AuNPs.

#### 3.4.2. HA Detection

**Figure 5:**
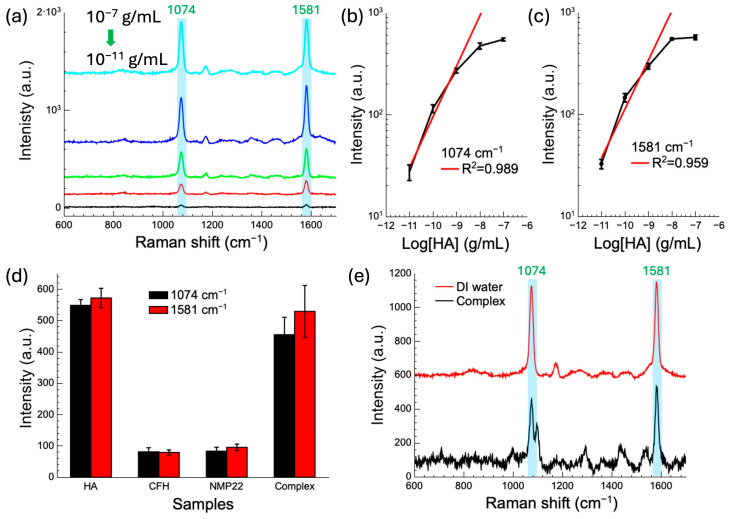
SERS-based detection of HA molecules. (**a**) Raman spectra of 4-MBA at HA concentrations ranging from 10^−7^ to 10^−11^ g/mL. (**b**,**c**) The relationship between the Raman intensity and HA concentration at 1074 cm^−1^ and 1581 cm^−1^, respectively. (**d**) Raman peak intensities at 1074 and 1581 cm^−1^ for the anti-HA-functionalized SERS substrate tested with HA, CFH, and NMP22 antigens in deionized water, as well as HA antigen in a simulated complex environment containing 10% FBS, each at a concentration of 10^−7^ g/mL. (**e**) Raman spectra of HA detection at 10^−7^ g/mL in a simulated complex environment containing 10% FBS, compared to HA detection in deionized water. Measurements were taken at five distinct locations for each condition.

Once the antibody-functionalized SERS substrate and 4-MBA SERS tags were successfully prepared, we proceeded with the detection of HA molecules. Starting with an initial HA concentration of 10^−7^ g/mL, we monitored the intensity changes of the characteristic Raman peaks of 4-MBA at 1074 cm^−1^ and 1581 cm^−1^, as shown in Figure 5a. Notably, 4-MBA and MG share common SERS peaks due to their similar aromatic ring structures. The peak at 1074 cm^−1^ corresponds to in-plane ring breathing and C–S bond stretching vibrations, while the peak at 1581 cm^−1^ arises from C–C stretching vibrations within the benzene ring [48]. The Raman intensity decreased progressively with a decreasing HA concentration, achieving a detection limit as low as 10^−11^ g/mL. Figure 5b,c illustrate the relationship between the Raman intensity and HA concentration at 1074 and 1581 cm^−1^, respectively, confirming the sensitivity and reliability of the detection method. The relationship between the Raman intensity and HA concentration is linear within the range of 10^−9^ to 10^−11^ g/mL. However, at higher HA concentrations, the Raman signal may reach saturation due to the limited AuNP surface area available for antibody conjugation on the SERS substrate, resulting in a nonlinear response as the concentration exceeds a certain threshold.

For the specificity testing of the SERS substrate functionalized with anti-HA antibodies, we selected FDA-approved bladder cancer biomarkers, namely human complement factor H-related protein (CFH) and nuclear matrix protein 22 (NMP22), as controls [49]. After immobilizing the anti-HA antibodies on the SERS substrate, 10^−7^ g/mL concentrations of the HA, CFH, and NMP22 antigens were separately applied to the system. The sandwich immunoassay configuration was employed, utilizing SERS tags composed of AuNPs conjugated with 4-MBA molecules and anti-HA antibodies. The specificity of this assay is based on the unique binding of antibodies to their corresponding antigens. When HA serves as the target antigen, a distinct antibody–antigen–antibody–Raman molecule structure forms, producing a strong and characteristic Raman signal. In contrast, non-specific binding with CFH or NMP22 antigens yields only background signals. As shown in Figure 5d, a comparison of the Raman peak values at 1074 and 1581 cm^−1^ for HA, CFH, and NMP22 highlights the excellent specificity of this method. Distinct Raman peaks are observed exclusively in the presence of HA, validating the SERS-based sandwich immunoassay as a highly selective method for HA detection.

To evaluate HA detection in real biological samples, we simulated a complex biological environment by combining 100 μL of 10^−6^ g/mL HA, 100 μL of 10% FBS, and 800 μL of deionized water. FBS, widely used in cell culture and assay development, contains a rich mixture of proteins, growth factors, and bioactive molecules that mimic physiological conditions, making it an excellent proxy for complex biological matrices. As shown in Figure 5d,e, the results illustrate the Raman peak values at 1074 and 1581 cm^−1^, along with the complete Raman spectra. HA detection at 10^−7^ g/mL exhibited weaker Raman signals and greater variability between the measurements in this complex environment compared to detection in deionized water. This reduction in signal strength and consistency is likely due to incomplete antigen–antibody binding caused by matrix interference in the complex environment. Matrix interference, commonly observed in biological samples, can result from several factors. The non-specific adsorption of biomolecules in the sample may occupy the detection surface or partially block antibody-binding sites, thereby reducing the efficiency of antigen–antibody interactions. Steric hindrance from large interfering substances in the matrix can further physically obstruct the binding of antigens to antibodies, limiting their accessibility and interaction. Additionally, alterations in the chemical environment, such as changes in the pH, ionic strength, or temperature, may impact the structural integrity or functional conformation of antigens and antibodies, weakening their binding affinity. These combined effects contribute to the observed decrease in the Raman signal intensity and variability in the measurements. Despite these challenges, the characteristic Raman peaks of HA remain detectable, demonstrating the robustness of the SERS-based detection system, even under complex biological conditions.

Our findings demonstrate that the antibody-functionalized SERS substrate is a highly effective platform for the detection of HA molecules, with detection limits as low as 10^−11^ g/mL. This level of sensitivity is well suited for clinical applications, as many studies suggest disease-specific HA concentration thresholds, such as 490–500 ng/mL for bladder cancer [38], 250 ng/mL for breast cancer [39], 101 ng/mL for upper gastrointestinal cancer [40], 50.13 ng/mL for prostate cancer [41], and 46.5 ng/mL for liver fibrosis in chronic liver disease [42]. The SERS-based detection system provides a powerful alternative to conventional methods, offering enhanced performance with simpler, cost-effective procedures. Furthermore, it requires less reliance on expensive instrumentation compared to traditional detection techniques, making it accessible for broader clinical use. Despite minor challenges, such as matrix interference in complex biological environments, the inherent advantages of SERS, such as high sensitivity, molecular specificity, and robust performance, make it a promising tool for early disease screening and point-of-care (POC) diagnostics [50,51]. Our SERS substrate enables the detection of HA below clinical thresholds, supporting early cancer detection, disease monitoring, and personalized medicine, while also extending its applicability to other low-concentration biomarkers. The fabrication process is compatible with flexible substrates [52,53], enabling the development of adaptable systems. Advances in low-cost, miniaturized Raman spectrometers further enhance their practicality for on-site testing. By integrating this SERS-based platform with compact and cost-effective Raman spectrometers, it is feasible to develop portable, user-friendly devices tailored for POC testing. Additionally, as shown in Table 2, immunoassay-based methods, such as ELISA [16], fluorescence-based immunoassays [21], LSPR-based refractive index immunoassays [18], and the current approach, achieve improved detection limits compared to other techniques for HA detection, including capillary electrophoresis, high-performance liquid chromatography (HPLC), electrochemistry, resonance Rayleigh scattering, phosphorescence, and fluorescence-based methods. Both the LSPR-based refractive index immunoassay and the current approach leverage the advantages of the localized surface enhancement mechanism and immunoassay, demonstrating promising performance. Overall, these advancements pave the way for the integration of SERS-based technologies into routine clinical practice, enhancing the early detection and monitoring of HA-associated diseases.

## 4. Conclusions

In this study, we successfully developed a highly sensitive and specific SERS-based immunoassay for biomarker detection, demonstrating its potential as a valuable tool for biomedical diagnostics. This platform leverages an inverted pyramid SU-8 nanostructured substrate, fabricated using nanoimprint lithography, which provides significant advantages in terms of uniformity, reproducibility, and scalability for large-scale production. The sharp apices and edges of the inverted pyramids, along with the uniform distribution of the AuNPs self-assembled on the APTES-functionalized surface, establish a configuration that is well suited for enhanced SERS analysis. The geometric design of the substrate effectively amplifies signals, playing a crucial role in achieving the ultra-sensitive detection capabilities of the system.

Performance testing of the SERS substrate using MG as the analyte demonstrated consistent and robust signal enhancement, achieving a detection limit as low as 10^−12^ M, with the enhancement factor calculated as 8.10 × 10^7^ for the 1613 cm^−1^ peak. This result confirms the suitability of the substrate for highly sensitive biomarker detection in subsequent applications. The platform incorporates functionalized antibodies for biomarker recognition and 4-MBA as the Raman reporter molecule, achieving detection limits as low as 10^−11^ g/mL for HA, a biomarker selected for its significant role in the progression of cancer and inflammation.

To evaluate the performance of the platform, a series of tests were conducted, including sensitivity and specificity assessments, reproducibility analysis, and detection in challenging biological environments, demonstrating its ability to distinguish HA from non-specific antigens, even in complex biological matrices. These results underscore the robustness and versatility of the SERS-based immunoassay in overcoming the limitations of traditional detection methods, such as low sensitivity and reliance on expensive equipment. The non-invasive nature of the system highlights its potential for early disease screening and applications in personalized medicine.

The antigen–antibody paradigm is highly specific, yet its relatively effort-intensive nature and reliance on a sandwich structure present opportunities for improvement. Potential alternatives include label-free detection leveraging the intrinsic capabilities of SERS [60], molecularly imprinted polymers (MIPs) for synthetic recognition [61], peptide-based binders, and aptamers as cost-effective, stable substitutes for antibodies. These approaches offer the potential to streamline the detection process.

Despite minor challenges, such as the variability in signals in complex biological samples, this study provides a solid foundation for the future advancement of SERS-based diagnostics. While further validation and optimization are needed, the platform shows great promise for enhanced early detection, therapeutic monitoring, and individualized treatment strategies in clinical settings.

## Figures and Tables

**Table 1:** Characteristic SERS peaks of MG.

Raman Shift (cm^−1^)	Peak Assignment	Reference
438	Phenyl-C-phenyl out-of-plane bending	[32]
528	Phenyl-C-phenyl out-of-plane bending	[33]
799	Ring C–H out-of-plane bending	[32,33,34]
913	Ring C–H out-of-plane bending or ring breathing	[32,33,34]
1170	Ring C–H in-plane bending	[32,33,34]
1214	Ring C–H rocking	[33,34]
1292	Ring C–C stretching	[33]
1365	N-phenyl stretching	[32,33,34]
1390	N-phenyl stretching, ring C–H in-plane bending, and ring C–C stretching	[33,34]
1589	Ring C–C stretching	[33,34]
1613	Ring C–C stretching	[32,33]

**Table 2:** Comparison of detection limits for HA across various analytical methods.

Method	Detection Limit (g/mL)	Reference
Capillary electrophoresis	10^−6^	[54]
Size-exclusion HPLC	1.2 × 10^−5^	[55]
HPLC with fluorescence detection	3.55 × 10^−6^	[56]
Electrochemistry	3.4 × 10^−8^	[57]
Resonance Rayleigh scattering	9.6 × 10^−8^	[58]
Phosphorescence	3 × 10^−8^	[59]
Fluorescence	6.9 × 10^−9^	[20]
Fluorescence-based immunoassay	2 × 10^−10^	[21]
ELISA	10^−9^	[16]
LSPR-based refractive index immunoassay	8.3 × 10^−12^	[18]
SERS-based immunoassay	10^−11^	this work

## Data Availability

The data presented in this study are available upon request from the corresponding author.

## Supplementary Materials

The following supporting information can be downloaded at: https://www.mdpi.com/article/10.3390/nano15010064/s1, Note S1: Background SERS spectrum from a nanostructured substrate with self-assembled AuNPs; Note S2: XPS spectra of the SERS substrate at different stages of functionalization; Note S3: Optimal 4-MBA concentration for labeling of AuNPs; Note S4: Optimal EDC/NHS concentration, AuNP size, and sandwich assembly time for preparation of 4-MBA-labeled SERS tags.

## References

[1] Matthews H.K., Bertoli C., de Bruin R.A. (2022). Cell cycle control in cancer. Nat. Rev. Mol. Cell Biol..

[2] Hanahan D. (2022). Hallmarks of cancer: New dimensions. Cancer Discov..

[3] Ferlay J., Colombet M., Soerjomataram I., Parkin D.M., Piñeros M., Znaor A., Bray F. (2021). Cancer statistics for the year 2020: An overview. Int. J. Cancer.

[4] Peng L., Wang Z., Stebbing J., Yu Z. (2022). Novel immunotherapeutic drugs for the treatment of lung cancer. Curr. Opin. Oncol..

[5] Desai A., Scheckel C., Jensen C.J., Orme J., Williams C., Shah N., Leventakos K., Adjei A.A. (2022). Trends in Prices of Drugs Used to Treat Metastatic Non–Small Cell Lung Cancer in the US From 2015 to 2020. JAMA Netw. Open.

[6] Zigrossi A., Hong L.K., Ekyalongo R.C., Cruz-Alvarez C., Gornick E., Diamond A.M., Kastrati I. (2022). SELENOF is a new tumor suppressor in breast cancer. Oncogene.

[7] Xu M., Peng R., Min Q., Hui S., Chen X., Yang G., Qin S. (2022). Bisindole natural products: A vital source for the development of new anticancer drugs. Eur. J. Med. Chem..

[8] Cao X., Gu Y., Li Z., Ge S., Mao Y., Gu Y., Lu D. (2023). A SERS microfluidic chip for ultrasensitive and simultaneous detection of SCCA and CYFRA21-1 in serum based on Au nanobowl arrays and hybridization chain reaction. Sens. Actuators B Chem..

[9] Fan D., Bao C., Liu X., Feng J., Wu D., Ma H., Wang H., Wei Q., Du B. (2019). Facile fabrication of visible light photoelectrochemical immunosensor for SCCA detection based on BiOBr/Bi2S3 heterostructures via self-sacrificial synthesis method. Talanta.

[10] Er E., Sánchez-Iglesias A., Silvestri A., Arnaiz B., Liz-Marzán L.M., Prato M., Criado A. (2021). Metal nanoparticles/MoS2 surface-enhanced Raman scattering-based sandwich immunoassay for α-fetoprotein detection. ACS Appl. Mater. Interfaces.

[11] Roointan A., Mir T.A., Wani S.I., Hussain K.K., Ahmed B., Abrahim S., Savardashtaki A., Gandomani G., Gandomani M., Chinnappan R. (2019). Early detection of lung cancer biomarkers through biosensor technology: A review. J. Pharm. Biomed. Anal..

[12] Medetalibeyoglu H., Kotan G., Atar N., Yola M.L. (2020). A novel sandwich-type SERS immunosensor for selective and sensitive carcinoembryonic antigen (CEA) detection. Anal. Chim. Acta.

[13] Fass L. (2008). Imaging and cancer: A review. Mol. Oncol..

[14] Wang H., Zhang Y., Zhang H., Cao H., Mao J., Chen X., Wang L., Zhang N., Luo P., Xue J. (2024). Liquid biopsy for human cancer: Cancer screening, monitoring, and treatment. MedComm (2020).

[15] Nakagawa H., Fujita M. (2018). Whole genome sequencing analysis for cancer genomics and precision medicine. Cancer Sci..

[16] Lokeshwar V.B., ÖBek C., Pham H.T., Wei D., Young M.J., Duncan R.C., Soloway M.S., Block N.L. (2000). Urinary hyaluronic acid and hyaluronidase: Markers for bladder cancer detection and evaluation of grade. J. Urol..

[17] Azab M.Y., Hameed M.F.O., Obayya S.S.A. (2023). Overview of Optical Biosensors for Early Cancer Detection: Fundamentals, Applications and Future Perspectives. Biology.

[18] Yang Z.-Y., Chang W.-H., Chiu Y.-C., Lin C.-H. (2023). Noninvasive Detection of Bladder Cancer Markers Based on Gold Nanomushrooms and Sandwich Immunoassays. ACS Appl. Nano Mater..

[19] Chen W.-Y., Lin C.-H., Chen W.-T. (2013). Plasmonic phase transition and phase retardation: Essential optical characteristics of localized surface plasmon resonance. Nanoscale.

[20] Zhang J., Feng H.-W., Chen H., Kang W., Xu X.-D. (2024). Highly Selective and Sensitive Detection of Hyaluronic Acid Based on a Pyrene-Cored Cationic Aggregation-Induced Emission Luminogen. Microchem. J..

[21] Martins J.R.M., Passerotti C.C., Maciel R.M.B., Sampaio L.O., Dietrich C.P., Nader H.B. (2003). Practical determination of hyaluronan by a new noncompetitive fluorescence-based assay on serum of normal and cirrhotic patients. Anal. Biochem..

[22] Wang L., Wang X., Cheng L., Ding S., Wang G., Choo J., Chen L. (2021). SERS-based test strips: Principles, designs and applications. Biosens. Bioelectron..

[23] Ho H.L., Yang J.Y., Lin C.H., Shieh J., Fang Huang Y., Ho Y.H., Ko T.S., Hsu C.C., Ostrikov K. (2023). Plasma-Etched Nanograss Surface without Lithographic Patterning to Immobilize Water Droplet for Highly Sensitive Raman Sensing. Adv. Mater. Interfaces.

[24] Chen Y.-J., Chang W.-H., Li C.-Y., Chiu Y.-C., Huang C.-C., Lin C.-H. (2021). Direct synthesis of monolayer gold nanoparticles on epoxy based photoresist by photoreduction and application to surface-enhanced Raman sensing. Mater. Des..

[25] Wang Z., Zong S., Wu L., Zhu D., Cui Y. (2017). Sers-Activated Platforms for Immunoassay: Probes, Encoding Methods, and Applications. Chem. Rev..

[26] Wei Q., Dong Q., Pu H. (2023). Multiplex Surface-Enhanced Raman Scattering: An Emerging Tool for Multicomponent Detection of Food Contaminants. Biosensors.

[27] Mathew E., Jenczyk J., Miłosz Z., Henzie J., Latsunskyi I., Florczak P., Andrzejewska W., Lewandowski M., Wiesner M. (2024). Polarized-SERS of non-isotropic molecules on thermally-induced corrugated plasmonic surface supporting a NIR-SPP mode. Appl. Surf. Sci..

[28] Yeh T.-W., Hung Y.-H., Chung C.-S., Yeh S.-J., Lee H.-Y., Lin C.-H. (2024). Nanotransfer Printed Dual-Layer Metasurfaces for Infrared Cut-off Applications. ACS Appl. Nano Mater..

[29] Chen Y.-J., Chang W.-H., Lin C.-H. (2020). Selective Growth of Patterned Monolayer Gold Nanoparticles on SU-8 through Photoreduction for Plasmonic Applications. ACS Appl. Nano Mater..

[30] Bastús N.G., Comenge J., Puntes V. (2011). Kinetically controlled seeded growth synthesis of citrate-stabilized gold nanoparticles of up to 200 nm: Size focusing versus Ostwald ripening. Langmuir.

[31] Laven P. (2021). MiePlot. http://www.philiplaven.com/mieplot.htm.

[32] Zhang Y., Huang Y., Kang Y., Miao J., Lai K. (2021). Selective recognition and determination of malachite green in fish muscles via surface-enhanced Raman scattering coupled with molecularly imprinted polymers. Food Control..

[33] Kumar P., Khosla R., Soni M., Deva D., Sharma S.K. (2017). A highly sensitive, flexible SERS sensor for malachite green detection based on Ag decorated microstructured PDMS substrate fabricated from Taro leaf as template. Sens. Actuators B Chem..

[34] Wicaksono W.P., Dang H., Lee S., Choo J. (2024). Electrochemical surface-enhanced Raman spectroscopy analysis of malachite green on gold substrates. Appl. Surf. Sci..

[35] Tammi M.I., Oikari S., Pasonen-Seppänen S., Rilla K., Auvinen P., Tammi R.H. (2019). Activated hyaluronan metabolism in the tumor matrix—Causes and consequences. Matrix Biol..

[36] Lokeshwar V.B., Öbek C., Soloway M.S., Block N.L. (1997). Tumor-associated hyaluronic acid: A new sensitive and specific urine marker for bladder cancer. Cancer Res..

[37] Morera D.S., Hennig M.S., Talukder A., Lokeshwar S.D., Wang J., Garcia-Roig M., Ortiz N., Yates T.J., Lopez L.E., Kallifatidis G. (2017). Hyaluronic acid family in bladder cancer: Potential prognostic biomarkers and therapeutic targets. Br. J. Cancer.

[38] El-Hefnawy A.S., Rizk E.M.A.E.A., Al Demerdash Khamis N.M., Barakat M.A.A., Khater S.M., Shokeir A.A. (2020). Urinary hyaluronic acid: A versatile marker of bladder cancer. Int. Urol. Nephrol..

[39] Peng C., Wallwiener M., Rudolph A., Ćuk K., Eilber U., Celik M., Modugno C., Trumpp A., Heil J., Marmé F. (2016). Plasma hyaluronic acid level as a prognostic and monitoring marker of metastatic breast cancer. Int. J. Cancer.

[40] Aghcheli K., Parsian H., Qujeq D., Talebi M., Mosapour A., Khalilipour E., Islami F., Semnani S., Malekzadeh R. (2012). Serum hyaluronic acid and laminin as potential tumor markers for upper gastrointestinal cancers. Eur. J. Intern. Med..

[41] Skarmoutsos I., Skarmoutsos A., Katafigiotis I., Tataki E., Giagini A., Adamakis I., Alamanis C., Duvdevani M., Sitaras N., Constantinides C. (2018). Hyaluronic acid and hyaluronidase as possible novel urine biomarkers for the diagnosis of prostate cancer. Med. Oncol..

[42] Orasan O.H., Ciulei G., Cozma A., Sava M., Dumitrascu D.L. (2016). Hyaluronic acid as a biomarker of fibrosis in chronic liver diseases of different etiologies. Clujul Med..

[43] Marinho A., Cláudia N., Salette R. (2021). Hyaluronic Acid: A Key Ingredient in the Therapy of Inflammation. Biomolecules.

[44] Lokeshwar V.B., Block N.L. (2000). Ha-Haase Urine Test: A Sensitive and Specific Method for Detecting Bladder Cancer and Evaluating Its Grade. Urol. Clin. N. Am..

[45] Cowman M.K., Lee H.G., Schwertfeger K.L., McCarthy J.B., Turley E.A. (2015). The Content and Size of Hyaluronan in Biological Fluids and Tissues. Front. Immunol..

[46] Kang D.Y., Kim W.S., Heo I.S., Park Y.H., Lee S. (2010). Extraction of Hyaluronic Acid (Ha) from Rooster Comb and Characterization Using Flow Field-Flow Fractionation (Flfff) Coupled with Multiangle Light Scattering (Mals). J. Sep. Sci..

[47] Simone P., Alberto M. (2015). Caution Should be Used in Long-Term Treatment with Oral Compounds of Hyaluronic Acid in Patients with a History of Cancer. Clin. Drug Investig..

[48] Zhang X.Y., Han D., Pang Z., Sun Y., Wang Y., Zhang Y., Yang J., Chen L. (2018). Charge Transfer in an Ordered Ag/Cu2s/4-Mba System Based on Surface-Enhanced Raman Scattering. J. Phys. Chem. C.

[49] Darwiche F., Parekh D.J., Gonzalgo M.L. (2015). Biomarkers for Non-Muscle Invasive Bladder Cancer: Current Tests and Future Promise. Indian J. Urol..

[50] Xu K., Zhou R., Takei K., Hong M. (2019). Toward Flexible Surface-Enhanced Raman Scattering (SERS) Sensors for Point-of-Care Diagnostics. Adv. Sci..

[51] Yadav S., Sadique M.A., Ranjan P., Kumar N., Singhal A., Srivastava A.K., Khan R. (2021). SERS Based Lateral Flow Immunoassay for Point-of-Care Detection of SARS-CoV-2 in Clinical Samples. ACS Appl. Bio Mater..

[52] Liang C.-C., Lin C.-H., Cheng T.-C., Shieh J., Lin H.-H. (2015). Nanoimprinting of Flexible Polycarbonate Sheets with a Flexible Polymer Mold and Application to Superhydrophobic Surfaces. Adv. Mater. Interfaces.

[53] Lin C.-H., Lin H.-H., Chen W.-Y., Cheng T.-C. (2011). Direct imprinting on a polycarbonate substrate with a compressed air press for polarizer applications. Microelectron. Eng..

[54] Chindaphan K., Wongravee K., Nhujak T., Dissayabutra T., Srisa-Art M. (2019). Online preconcentration and determination of chondroitin sulfate, dermatan sulfate and hyaluronic acid in biological and cosmetic samples using capillary electrophoresis. J. Sep. Sci..

[55] Suárez-Hernández L.A., Camacho-Ruíz R.M., Arriola-Guevara E., Padilla-Camberos E., Kirchmayr M.R., Corona-González R.I., Guatemala-Morales G.M. (2021). Validation of an Analytical Method for the Simultaneous Determination of Hyaluronic Acid Concentration and Molecular Weight by Size-Exclusion Chromatography. Molecules.

[56] Harmita H., Hayun H., Geofani M.H. (2020). Quantification of hyaluronic acid and methylsulfonylmethane in dietary supplements. Int. J. Appl. Pharm..

[57] Perk B., Büyüksünetçi Y.T., Anık Ü. (2023). Gold nanoparticle deposited electrochemical sensor for hyaluronic acid detection. Chem. Pap..

[58] Luo H.Q., Li N.B., Liu S.P. (2006). Resonance Rayleigh scattering study of interaction of hyaluronic acid with ethyl violet dye and its analytical application. Biosens. Bioelectron..

[59] Li D., Qin J., Lv J., Yang J., Yan G. (2018). “Turn on” room-temperature phosphorescent biosensors for detection of hyaluronic acid based on manganese-doped ZnS quantum dots. RSC Adv..

[60] La Verde G., Sasso A., Rusciano G., Capaccio A., Fusco S., Mayol L., Biondi M., Silvestri T., Netti P.A., La Commara M. (2023). Characterization of Hyaluronic Acid-Coated PLGA Nanoparticles by Surface-Enhanced Raman Spectroscopy. Int. J. Mol. Sci..

[61] Vaneckova T., Bezdekova J., Han G., Adam V., Vaculovicova M. (2020). Application of molecularly imprinted polymers as artificial receptors for imaging. Acta Biomater..

