# Supplementary material for: Inverted Pyramid Nanostructures Coupled with a Sandwich Immunoassay for SERS Biomarker Detection
Source: Nanomaterials (Basel). 2025 Jan 2;15(1):64. doi: 10.3390/nano15010064 (PMC11722957; doi:10.3390/nano15010064)
Supplement: Supplementary file 1 [file nanomaterials-15-00064-s001.zip › nanomaterials-3367427-supplementary.pdf]

Supporting Information

Inverted Pyramid Nanostructures Coupled with a Sandwich Immunoassay for SERS Biomarker Detection

Wen-Huei Chang [1,*], Shao-Quan Zhang [2], Zi-Yi Yang [2] and Chun-Hung Lin [2,3,4,*]

[1] Department of Applied Chemistry, National Pingtung University, Pingtung 90003, Taiwan
[2] Department of Photonics, National Cheng Kung University, Tainan 70101, Taiwan
[3] Meta-nanoPhotonics Center, National Cheng Kung University, Tainan 70101, Taiwan
[4] Program on Key Materials, Academy of Innovative Semiconductor and Sustainable Manufacturing, National Cheng Kung University, Tainan 70101, Taiwan
* Correspondence: whchang@mail.nptu.edu.tw (W.-H.C.); chlin@ncku.edu.tw (C.-H.L.)

Note S1. Background SERS spectrum from a nanostructured substrate with self-assembled AuNPs

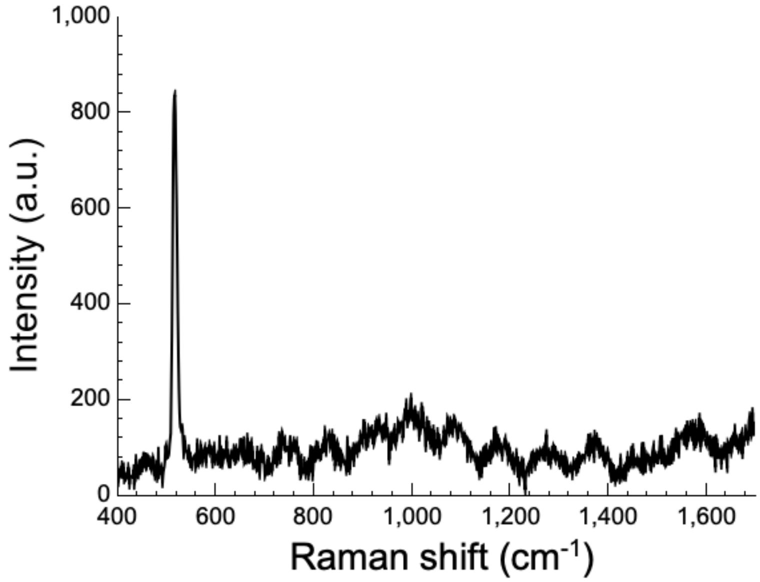

Figure S1. Background SERS spectrum from a nanostructured substrate with self-assembled AuNPs, showing the average intensity measured across five distinct locations. The Raman peak at 518 cm^{-1} is attributed to the silicon substrate.

Note S2. XPS spectra of the SERS substrate at different stages of functionalization

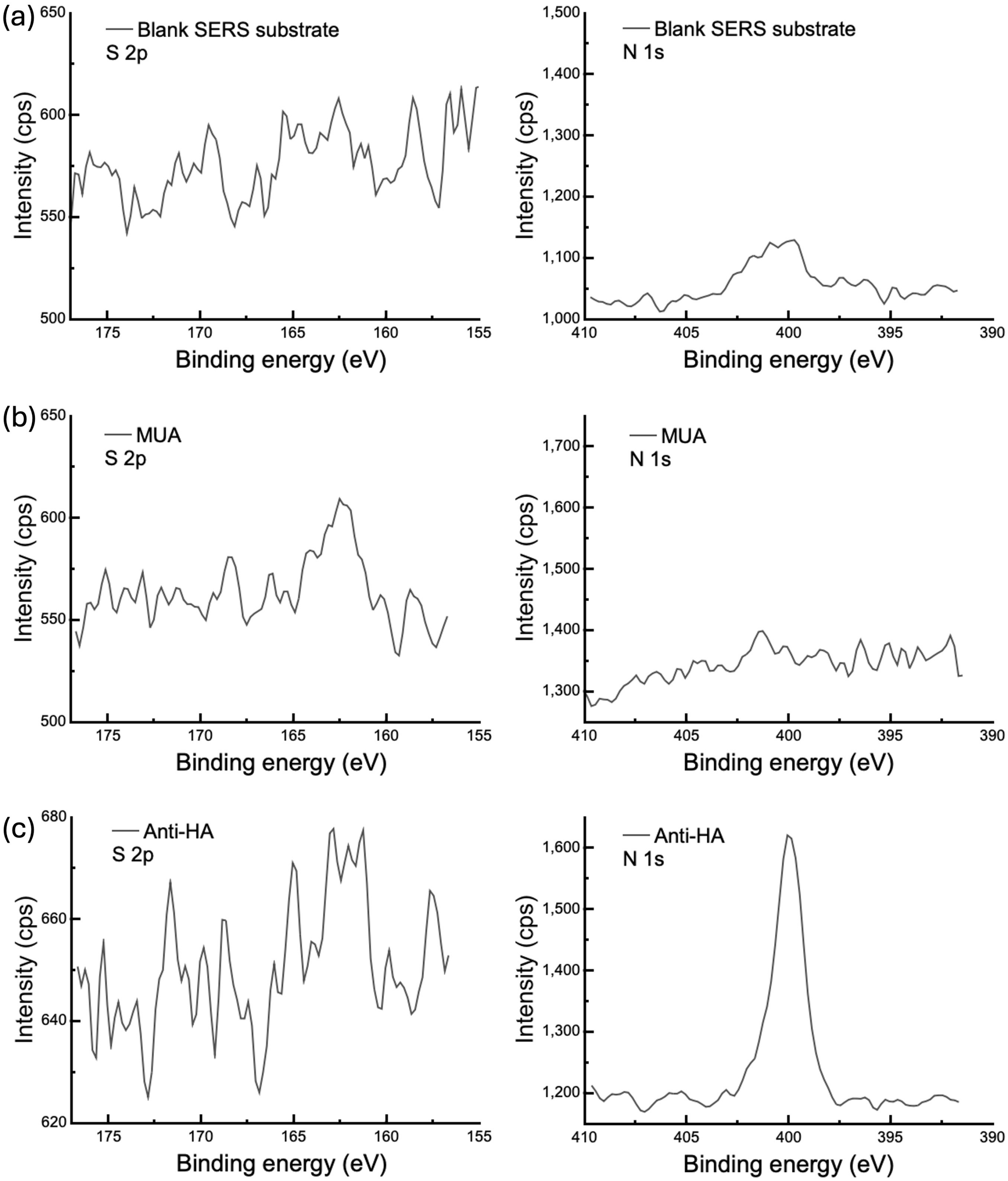

Figure S2. XPS spectra of the SERS substrate at different stages of functionalization: (a) the blank substrate, (b) after modification with MUA, and (c) after modification with anti-HA antibodies.

Note S3: Optimal 4-MBA concentration for labeling AuNPs

The addition of a small amount of TWEEN 20 to the AuNP solution effectively increases the spacing between negatively charged AuNPs, reducing the likelihood of aggregation. As illustrated in the absorption spectra shown in Figure S3, AuNPs labeled with varying concentrations of 4-MBA display varying degrees of aggregation. At a 4-MBA concentration of 10^{-2} M, the absorption ratio at 520 nm to 650 nm falls below 1, indicating significant aggregation. Although the ratio exceeds 1 at 10^{-3} M, it still indicates a tendency for aggregation, posing challenges for subsequent antibody modifications. To achieve greater stability during the labeling process, a 4-MBA concentration of 10^{-4} M was chosen as optimal.

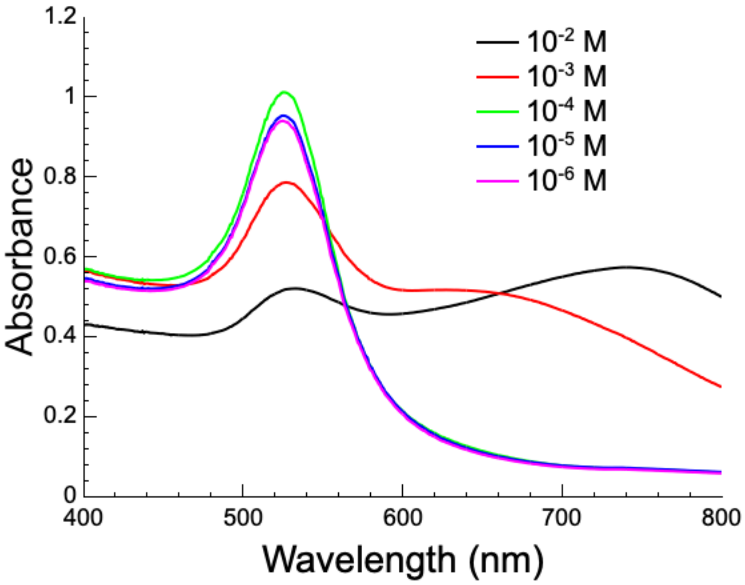

Figure S3. Absorption spectra of 4-MBA-labeled AuNPs with a diameter of approximately 37.4 nm, labeled using 4-MBA concentrations ranging from 10^{-2} to 10^{-6} M.

Note S4: Optimal EDC/NHS concentration, AuNP size, and sandwich assembly time for preparing 4-MBA-labeled SERS tags

The addition of TWEEN 20 to the AuNP solution during the modification with Raman reporter molecule 4-MBA increases the energy required for carboxyl group activation. To address this, 10 μL of EDC/NHS (volume ratio 1:1.5) at varying concentrations was used to activate the carboxyl groups on 4-MBA molecules and conjugate them with anti-HA antibodies. BSA was employed to fill any unoccupied spaces on the AuNP surface. The functionalized SERS tags, containing 4-MBA and anti-HA, were directly applied onto SERS substrates pre-modified with antibodies and antigens (HA at a concentration of 10^{-8} g/mL) in a sandwich format to facilitate specific antibody-antigen binding, followed by Raman detection.

As shown in Figure S4a, the Raman intensity of the 4-MBA characteristic peak at 1074 cm^{-1} was strongest when EDC/NHS was employed at a concentration of 350 mM (volume ratio 1:1.5). Lower concentrations of EDC/NHS likely resulted in insufficient carboxyl group activation, leading to incomplete anti-HA modification and fewer functional antibodies available to bind to antigens at the SERS substrate, thus weakening the signal. At concentrations exceeding 350 mM, partial aggregation of AuNPs reduced their enhancement activity, diminishing the Raman signal. Therefore, a concentration of 350 mM EDC/NHS was selected as the optimal condition for stabilizing antibodies through carboxyl activation.

Subsequently, various AuNP sizes were modified using 350 mM EDC/NHS (volume ratio 1:1.5) and tested with HA at 10^{-8} g/mL. As shown in Figure S4b, AuNPs with a diameter of approximately 37.4 nm achieved the best results, whereas those with a larger diameter (approximately 52.8 nm) exhibited weaker intensity. This is likely because larger nanoparticles generate fewer hotspots per unit area in the sandwich structure, weakening the localized surface plasmon resonance (LSPR) effect.

Finally, the sandwich assembly time for incubating 50 μL of SERS tags with the antigen-antibody-functionalized SERS substrate was optimized to evaluate its effect on Raman intensity. As shown in Figure S4c, incubation times of 2, 4, 6, and 8 hours were assessed. At 2 hours, the 4-MBA-labeled SERS tags had not fully immobilized on the substrate. The Raman signal reached its maximum at 4 hours and plateaued thereafter, indicating that the substrate had reached saturation.

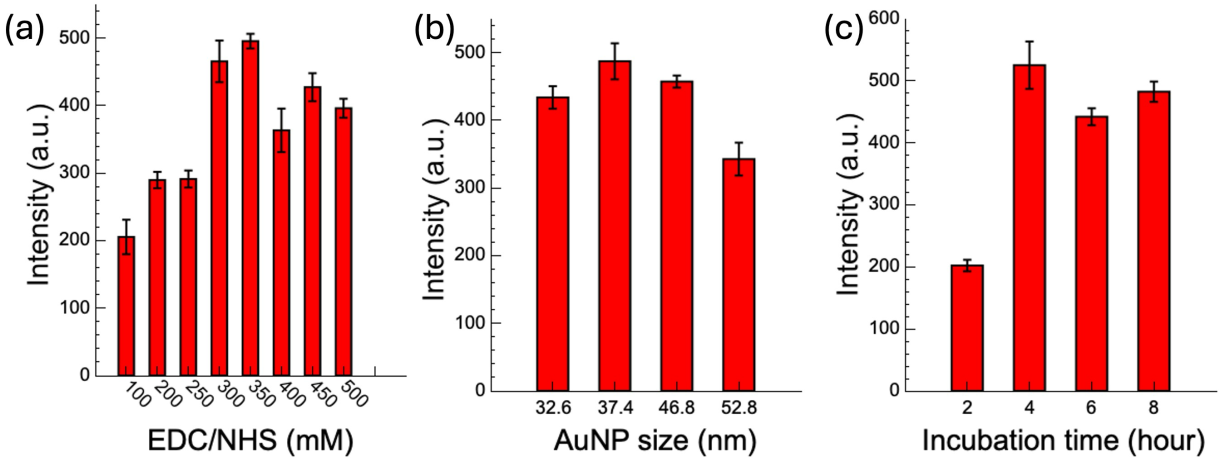

Figure S4. Raman intensity at 1074 cm^{-1} for (a) varying EDC/NHS concentrations (100 to 500 mM), (b) AuNP sizes (approximately 32.6, 37.4, 46.8, and 52.8 nm), and (c) sandwich assembly times (2, 4, 6, and 8 hours) during the preparation of 4-MBA-labeled SERS tags. Unless otherwise specified, the standard preparation parameters used were an EDC/NHS concentration of 350 mM, an AuNP size of approximately 37.4 nm, and a sandwich assembly time of 4 hours. The Raman intensities represent the average measured across five distinct locations.
